# Case of Poisoning by Laudanum

**Published:** 1843-07-08

**Authors:** J. L. Burt

**Affiliations:** Blackwood Town, N. J.


### CASE OF POISONING BY LAUDANUM.

BY J. L. BURT, M. D.

To the Editor of the Examiner.

*Dear Sir*,—I present for publication, should you think it of sufficient importance, a case of poisoning by laudanum, which terminated fatally. It occurred in a child, whose mother had been in the habit of forcing sleep, by means of Godfrey's cordial. I send you a specimen of the laudanum taken, and such as is generally kept in the stores here. This was given by mistake for the cordial. I should be much obliged to you if you can give me the relative strength of this specimen, and that of the officinal tinct. opii.

#### CASE.

I was called upon at one, A. M., of the 15th June, to see John Budd, aged thirteen weeks. At half past seven o'clock, of the previous evening, its mother had given it a tea-spoonful and half, of what she supposed was Godfrey's cordial, in order to procure sleep. It slept soundly, and no particular attention was called to its state, until the mother was waked about one, A. M., by a fit, as she called it. In a few minutes she went down stairs, and discovered that she had given laudanum. I was sent for at this time, about five hours and three quarters from the administration of the dose.

*Found.* Face and head flushed and hot, with great turgescence; limbs drawn up; eyes in constant and rapid motion, thrown upwards; iris contracted so much as to be scarcely perceptible; and eyelids immovable; no apparent vision; the utterance of its name in a loud key seemed barely to be heard. Pulse 180, small and thready. Deglutition very difficult; stertorus breathing; tongue flabby and pale. Extremities cold and discolored.

*Treatment*—Not having a stomach pump at hand it was not tried; I am inclined to think that it would have been useless, or almost impossible to apply it owing to the closeness of the mouth, and the resistance which it afforded upon any attempt to open it. ℞. Sulph. zinc., grs. xv., in solution. In fifteen minutes. ℞. grs. v., repeated at the same interval, grs. v. twice more, followed by grs. xv. No vomiting produced—directed an attendant to cool her hand upon the bricks, and apply it suddenly to the abdomen. After several trials a very moderate emesis was procured. Encouraged by warm drink without effect. No visible sign of laudanum in the matter discharged. Appeared to be medicine taken, and some indigested milk. Had, during this period, applied sinapisms to the extremities. Warm bath to the thighs, and continued friction. ℞. Carb. Ammon., grs. v., repeated at intervals of twenty minutes, in order to decompose any opium which might remain in the stomach, and diminish the solubility of the morphine.

Seemed in a short time somewhat relieved from the comatose symptoms. Continued efforts to procure emesis in vain; coma as profound as before; kept up constant motion and friction.

Six and a half, A. M. Pupils rather more dilated; extremities cold, and of a darker tinge. Pulse almost imperceptible; deglutition impracticable.

Eleven, A. M. Coma more profound; pulse fifty, full, soft, intermittent. Continue friction as before.

Twelve and a half, P. M. Dead. Countenance and skin generally discoloured; body bloated; pupils very much contracted. No post mortem was allowed. Thus death occurred in seventeen hours from the administration of the dose.

*Blackwood Town, N. J.* June 16, 1843.

